# Biocompounds Content Prediction in Ecuadorian Fruits Using a Mathematical Model

**DOI:** 10.3390/foods8080284

**Published:** 2019-07-25

**Authors:** Wilma Llerena, Iván Samaniego, Ignacio Angós, Beatriz Brito, Bladimir Ortiz, Wilman Carrillo

**Affiliations:** 1 Facultad de Ciencia e Ingeniería en Alimentos y Biotecnología, Universidad Técnica de Ambato (UTA), Av. Los Chasquis y Río Payamino, 180103 Ambato, Ecuador; 2 Facultad de Ciencias Pecuarias, Ingeniería en Alimentos, Universidad Técnica Estatal de Quevedo, Km 7 1/2 vía Quevedo-El Empalme, 120313 Los Ríos, Ecuador; 3 Departamento de Nutrición y Calidad, Instituto Nacional de Investigaciones Agropecuarias (INIAP), Panamericana Sur Km. 1, 170516 Mejía, Ecuador; 4 Departamento de Investigación, Universidad Técnica de Babahoyo, Av. Universitaria Km 2 1/2 Av. Montalvo, 120301 Babahoyo, Ecuador

**Keywords:** chemometrics, mathematical model, metaheuristic techniques, color, araza, blackberry, Andean blueberry, naranjilla, tamarillo, goldenberry

## Abstract

Anthocyanins, carotenoids and polyphenols are biomolecules that give the characteristic color to fruits. Carotenoids relate to yellow, orange and red colors whereas anthocyanins and polyphenols mainly relate to purple and red colors. Presently, standard determination of antioxidants is carried out using relatively complex methods and techniques. The aim of this study was to develop a mathematical prediction model to relate the internal color parameters of the Amazonic fruits araza (*Eugenia stipitata* Mc Vaugh), Andean fruit blackberry (*Rubus glaucus* Benth), Andean blueberry (*Vaccinium floribundum* Kunth), goldenberry (*Physalis peruviana* L.), naranjilla (*Solanum quitoense* Lam.), and tamarillo (*Solanum betaceum* Cav.) to their respective anthocyanins, carotenoids and polyphenols contents. The mathematical model was effective in predicting the total anthocyanins content (TAC), the total carotenoids content (TCC) and finally the total phenolic content (TPC) of fruits assayed. Andean blueberry presented a TPC with an experimental value of 7254.62 (mg GAE/100 g sample) with respect to a TPC prediction value of 7315.73 (mg GAE/100 g sample). Andean blackberry presented a TAC with an experimental value of 1416.69 (mg chloride cyanidin 3-glucoside/100 g) with respect to a prediction TAC value of 1413 (mg chloride cyanidin 3-glucoside/100 g).

## 1. Introduction

Due to its geographical location, Ecuador is a diverse country in terms of climate and fruit production. Fruit consumption is clearly associated with health benefits such as enhancing the immunologic system, reduction of cellular oxidative damage and protection against cancer development [1]. These properties are attributed to the presence of phytochemicals and nutrients with antioxidant properties [2,3,4]. Antioxidants can have chemoprotective effects, which include prevention of cardiac diseases, antidiabetic activity and vasoprotective properties [5,6,7].

Antioxidants can be classified in four phytochemicals main groups: phenolic compounds, including anthocyanins, terpene substances, including carotenoids, sulphur compounds, and finally, nitrogen compounds alkaloids. Amongst them, the first three groups are the most important as bioactive constituents in fruits are responsible for skin and pulp color [8].

Polyphenols are responsible for the red, blue and purple colors in many fruits and legumes [9,10]. In this group, anthocyanin is a group of water-soluble pigments, composed by a molecule of anthocyanidin, also named aglycone, linked to a sugar with a β-glucosides bond [1]. Its color and stability depend on several factors such as chemical structure, pH and temperature [5]. Anthocyanins are associated with flavylium cation, which produces red color at low pH ≤ 1.0 [1]. Concerning carotenoids, these compounds are common natural pigments. Around 600 carotenoids have been described in the literature, β-carotene being the most representative. Carotenoids pigments are responsible for red, orange, and yellow hues of plant leaves, fruits, and flowers, as well as the colors of some birds, insects, fish, and crustaceans. Plants, bacteria, fungi, and algae can synthesize carotenoids. However, animals and humans incorporate carotenoids through their diet. Some carotenoids can be used as a source of vitamin A [11].

During ripening, a series of biochemical and physiological processes occur, producing changes in the texture, flavor and color of the fruits [12,13]. Color changes are evident during fruit development and ripening and keep on going after harvesting. Orange color becomes evident in β-carotene rich fruits as araza, naranjilla, tamarillo and goldenberry fruits, when the degreening process occurs. During the maturation stage, carotenoids build up and at the same time, chlorophylls start a degradation process to a pheophytin form [13,14]. Fruits of deep red and blue colors such as blackberry and Andean blueberry show a superficial color change due to an accumulation of anthocyanins associated with changes in the concentration of sugars and organic acids [15].

Determination of fruit properties is of utmost importance in the food industry, color being the most important of visual attributes. Consumers use color as a fast index of quality of a fruit. Color is associated with its taste, freshness and nutritional value. Moreover, color is related to fruit shelf life: a reduction in its characteristic traits can be easily associated with decay [16,17,18].

Color and antioxidant content can be quantified based on the characteristic radiation wavelength absorption of each pigment in the visible region of the electromagnetic spectrum [19,20]. The classical spectrophotometric methods for determination of light absorption are carried out with equipment and specialized procedures, not easily affordable for small-medium scale enterprises. Nevertheless, quality can be related to color in an easier way, using portable spectrophotometric methods associated to a wide gamut color space L* a* b* coordinates [21,22,23]. From these measurements, non-destructive chemometric prediction methods can be created using deterministic and stochastic mathematical models. These models are fast, precise and easy enough to be used in routine food quality control [24,25,26].

The aim of the present work was to determine the total content of anthocyanins, carotenoids and polyphenols, of six representative Andean and tropical fruits from Ecuador using the UV-visible spectrophotometry and to develop a mathematical tool to predict the nutritional value based on the measurement of the internal color, as a cheap and fast alternative quality method of analysis. Antioxidant activity was also determined.

## 2. Materials and Methods

### 2.1. Raw Material

Two tropical fruits, and four Andean fruits were chosen in this research. Araza (*Eugenia stipitata* Mc Vaugh) is a fruit from the genus *Mirtaceae* cultivated all around the Amazonic basin. Two clones (INIAP 001 and INIAP 003) from the Orellana province in Ecuador were selected for their special aptitude to be industrially processed due to their unique flavor, strong sourness and short shelf life.

Blackberry (*Rubus glaucus* Benth) is a fruit native from the high lands of the intertropical region [27]. Cultivar ‘INIAP Andimora 2013′ is an improved clone cultivated in the Tungurahua province. This clone is thornless, has a high yield, high fruit quality and improved resistance to the main diseases affecting this plant.

Mortiño or Andean blueberry (*Vaccinium floribundum* Kunth) is a small perennial bush growing wild in the highlands in the Andes. Fruits are spherical berries of dark blue color, traditionally harvested in the Ecuadorian provinces of Bolivar, Cotopaxi, and Pichincha.

Naranjilla (*Solanum quitoense* Lam.) belongs to the Solanaceae family, native of the Andean medium ranges of Ecuador, Colombia and Central America [28]. The Instituto Nacional de Investigaciones Agropecuarias (INIAP) from Ecuador, through the National Program of Fruticultura, has generated technologies that allow generating resistant materials and practices of integrated agronomic management. One of these materials is the juicy naranjilla INIAP Quitoense 2009, which comes from a selection of the variety ‘Baeza’ years 2005–2007, and presents better characteristics in terms of vigor, yield capacity, productivity and physicochemical quality of fruits.

Tamarillo (*Solanum betaceum* Cav.) is a Solanaceae with a medium-sized fruit, oval berry, with a juicy bitter-sweet pulp. Ecotype ‘Anaranjado Gigante’ comes originally from the Tungurahua province and is mainly featured by its light orange pulp color [29].

Goldenberry (*Physalis peruviana* L.) is an annual or short-lived culture belonging to the genus *Solanaceae,* which is mostly grown for its ecotype ‘Golden Kenyan’ in the Tungurahua province of Ecuador. The fruit, usually commercialized with its distinctive protector calyx, is small, round and yellow with a medium sourness flavor [30].

### 2.2. Sample Preparation

Fifteen kilograms of each fruit was recollected from trees to ensure the heterogeneity. The fruits were washed with drinking water to reduce the microbial load, dirt and organic matter. They were then separated in portions of 1 kg to obtain 15 samples for each fruit assayed. Then, the maturity index was determined, and the fruit was homogenized, screened and stored in high-barrier plastic bags with hermetic seals at −18 °C, out of oxygen and light. The determination of internal color, anthocyanins, and total polyphenols was carried out taking into consideration 15 samples per fruit, in triplicate (*n* = 45 for each fruit). The total carotenoid content determination was carried out in duplicate (*n* = 30 for each fruit). The total flavonoids content was determinate only in three fruits (araza, naranjilla and tamarillo) and was made for triplicate (*n* = 45). All methods were validated using CV Horwitz—15 samples for fruits were used and duplicate and triplicate measurements were taken. CV Horwitz were considered significant with values ≤16.0%.

### 2.3. Physicochemical Analysis

#### 2.3.1. Texture

Fruit firmness was determined by puncture with two penetrometers Gullimex (Borne, Netherlands), using different probe diameters. The results were expressed in Newtons (N). Araza, naranjilla and tamarillo were tested using a FT327 model with a cylindrical probe of 6-mm diameter. Goldenberry, blackberry and Andean blueberry were tested with a FT011 model with a cylindrical probe of 2-mm diameter.

#### 2.3.2. Maturity Index

The maturity index (MI) was calculated based on the titratable acidity and the content of total soluble solids. Titratable acidity was obtained by acid-base neutralization according to Ecuadorian standards. The result was expressed in terms of percent of malic acid for araza [31] and blackberry [12] and citric acid for Andean blueberry [32], tamarillo [33], naranjilla [34], and goldenberry [35].

Total soluble solid (TSS) content was directly measured in the pulp of each sample with a digital handheld refractometer Atago, model PAL, 0–53 °Brix (Tokio, Japan). The results were expressed as g of sucrose per 100 g of sample (°Brix).

The MI was calculated with Equation (1), Tehranifar et al. (2010) [36].

MI = TSS/TA(1)

where,

MI: maturity index (dimensionless)
TSS: total soluble solid content (°Brix)
TA: titrable acidity (g/100 g)

#### 2.3.3. Internal Color

The internal color was determined with a handheld colorimeter ColorTec-PCM (ColorTec, Clinton, NJ, USA) with a measurement angle of 10°, D65 illuminant and 8 mm aperture. Chromatic properties of the fruit pulps were expressed in the CIE (Commission Internationale de l’Eclairage) L*a*b* color space in terms of coordinates L* luminosity, a* red/green and b* blue/yellow. From each fruit, 500 mL of pulp was extracted and homogenized. Samples of 30 mL were carefully poured in a Petri dish avoiding lumps or bubbles. The Petri dish was placed over a white surface and divided into four equal areas. Duplicate measurements were done in each quarter and center of the plate.

### 2.4. Total Anthocyanins Content (TAC)

TAC was determined using the differential pH method used by Rapisarda et al. (2000) [37]. The extraction was done with a magnetic stirrer for 60 min, taking 0.25 g of freeze dried sample, adding 10 mL of buffer solution at pH 1.0 (potassium chloride 0.2 N and hydrochloric acid 0.2 N) and a buffer solution at pH 4.5 (sodium acetate 1 M, hydrochloric acid 1 N). After centrifugation of the extract at 5000 rpm, 1 mL of the solution was diluted with the buffer solution to 10^−3^ for buffer pH 1.0 and 10^−1^ for buffer pH 4.5. The absorbance was measured in the supernatant and buffer solutions at 510 nm and 700 nm with a UV-VIS spectrophotometer Shimadzu, model 2200 (Shimaszu, Kioto, Japan). Results were expressed as mg of cyanidin-3-glucoside chloride/100 g of the dry weight sample (DW). TAC was calculated based on the following equation:

TAC = A × MW × DF × 100/Ɛ × W

where A is the absorbance, MW molecular weight of cyanidin-3-glucoside chloride (C_21_H_21_ClO_11_, 484.84 g/moL), DF is dilution factor, ε molar absorptivity (34,300), W = sample weight (g).

### 2.5. Total Carotenoids Content (TCC)

TCC was measured in the absence of light and oxygen, using 0.6–1.0 g of the freeze-dried sample. The extraction was done using 50 mL of a solvent mixture composed by hexane 50%, ethanol 25%, acetone 25% (*v/v/v*), 0.1% of butylated hydroxytoluene (BHT) (p/v) and 5 g of calcium chloride (p/v). These elements were added gradually. The mixture was mixed for 20 min in a refrigerated water bath at 4 °C. The phase separation was achieved by adding 15 mL of distilled water for 10 min. The extract was filtered and transferred to a separating funnel. The organic phase was transferred to a volumetric flask. Hexane was added to reach 50 mL. The determination of total carotenoids content was made using the UV-VIS spectrophotometer Shimadzu, model 2200 (Kioto, Japan) at 450 nm, using the method of Leong & Oey (2012) [38]. The results were expressed in terms of µg of β-carotene/µg of dry weight basis (DW). TCC was calculated based on the following equation:

TCC= A × VT × 10^4^/2592 × W

where A is the absorbance at 450 nm, VT is the volume total, 2592 is the coefficient of extinction molar of β-carotene in hexane and W is the weight of the sample and 10^4^ constant of conversion of units of µg/g.

### 2.6. Total Polyphenols Content (TPC)

TPC was determined following a method by Georgé et al. (2005) [39]. The extraction was made using a solution of 70% acetone (*v/v*) under magnetic stirring for 45 min, using 0.3–1.0 g of freeze-dried samples. The mixture was centrifuged for 10 min at 3500 rpm and the supernatant raw extract was recovered in Eppendorf vials. TPC A non-soluble fraction and the soluble compound TPC B fraction were evaluated from the raw extract. For the A fraction, a series of solutions of 25, 50 and 75 µL of the raw extract in 500 mL of pure methanol were prepared. The separation of soluble compounds was done by solid phase extraction (SPE) using C18 OASIS cartridges (Waters Corp.; Milford, MA, USA) previously conditioned following the fabricant instructions. 500 µL of raw extract were diluted in 3.5 mL of distilled water and a 2 mL aliquot of this solution was injected into the OASIS cartridge.

The quantification of A and B fractions was done using the Folin-Ciocalteu method with a UV-VIS spectrophotometer Shimadzu 2200 (Kioto, Japan) at 760 nm and both fractions were used (Abs_B_-Abs_A_) to corrected interferences. Gallic acid was used as standard. The curve was stablished (y = 0.0011x + 0.0529, R² = 0.9997). The total polyphenol content was expressed in terms of mg of gallic acid equivalents GAE/100 g of the DW sample.

### 2.7. Mathematical Modeling

Mathematical modeling was carried out using chemometric techniques of pattern recognition by object representation in a multidimensional space towards a reduced dimensionality space [25]. The correlation matrix between dependent and independent variables was determined using a multidimensional regression and the mean square method (Equations (2)–(4)). Finally, a results matrix was obtained (Table 1).

(2) $$A\overset{N}{\underset{K=1}{\sum }}x^{4}k\;+\;B\overset{N}{\underset{K=1}{\sum }}x^{3}k+C\overset{N}{\underset{K=1}{\sum }}x^{2}k=\;\overset{N}{\underset{K=1}{\sum }}x^{2}kYk$$

(3) $$A\overset{N}{\underset{K=1}{\sum }}x^{3}k\;+\;B\overset{N}{\underset{K=1}{\sum }}x^{2}k+C\overset{N}{\underset{K=1}{\sum }}xk=\;\overset{N}{\underset{K=1}{\sum }}xkYk$$

(4) $$A\overset{N}{\underset{K=1}{\sum }}x^{2}k\;+\;B\overset{N}{\underset{K=1}{\sum }}xk+CN=\;\overset{N}{\underset{K=1}{\sum }}Yk$$

In Table 1, color coordinates L*, a* and b* were the independent variables. TAC, TCC and TPC acted as the dependent variables for the n samples.

The robustness analysis of the mathematical prediction model was evaluated using the coefficient of determination. The data homogeneity was evaluated using the experimental residual analysis vs the predicted values. Only samples with standard deviations lower than 2σ were used [17]. The partial coefficients of the regression model were tested for signification (Equation (5)) to verify if parameters L*, a* and b* added value to the prediction model.

(5) $$t=\;\frac{bi}{\sqrt{Pij*S^{2}}}$$

where,

*bi*: partial regression coefficient
*Pij*: i-row and j-column of the reverse square sum and cross-product matrix
*S*^2^: estimator for the variance of the standard deviations and residues

The magnitude of the correlations between the variables was evaluated with the values of the determination coefficient (R^2^). The most influential variables in the model and the data with the better adjustments were used for a new multiple regression analysis, establishing the final mathematical model (Equation (6)).

(6) $$Y_{c}=\;b_{0}+\;b_{1}X_{1}+\;b_{2}X_{2}+\;b_{3}X_{3}$$

where,

*Y_c_*: the content of total antioxidant: anthocyanins, carotenoids and polyphenols
*b*_1_, *b*_2_, *b*_3_: regression coefficients
*X*_1_, *X*_2_, *X*_3_: color parameters L*, a* and b*

### 2.8. ABTS Assay

The extracts of araza (*Eugenia stipitata* McVaugh), tamarillo (*Solanum betaceum* Cav.) and naranjilla (*Solanum quitoense* Lam.) were used to evaluate their antioxidant activity using the ABTS method described by Piñuel et al. (2019). Trolox was used as the reference standard (0–800 µmoL Trolox/L). The curve was established (y = 0.0007x + 0.0671, R² = 0.999). The results obtained were expressed as µmol Trolox Equivalents TE/ g sample. All assays were made in triplicate [40].

### 2.9. DPPH Assay

The extracts of araza (*Eugenia stipitata* McVaugh), tamarillo (*Solanum betaceum* Cav.) and naranjilla (*Solanum quitoense* Lam.) were used to evaluate the antioxidant activity using the DPPH method described by Piñuel et al. (2019). Trolox was used as the reference standard (50–500 µmol Trolox/L) and the curve was established (y = 0.0013x + 0.007, R² = 0.999). The results obtained were expressed as µmol TE/g sample. All assays were made in triplicate [40].

### 2.10. Statistical Analysis

The results obtained in this study were presented as means ± standard deviation (SD). Differences between group values were determined using the one-way ANOVA analysis, followed by the Tukey’s test. All tests were considered with statistical differences at *p* < 0.05 and *p* < 0.01 using the software Graph Pad Prism 4. Moreover, the data obtained with the mathematical model were processed with the Statistica 10.0 software to obtain the analysis graphs.

## 3. Results and Discussion

### 3.1. Maturity Index

Climacteric fruits as araza, naranjilla and tamarillo can reach full ripening after harvesting. These fruits were harvested at physiological maturity. The MI is a parameter commonly used to establish the commercial maturity of these products [12]. As shown in Table 2, naranjilla and tamarillo presented an MI over 2.5 and 4.5 respectively. Araza is extremely perishable. The fruit used in this work, presented an MI between 1.15 and 2.05, corresponding to a partially mature green-yellow and over ripened full yellow color [41].

Non climacteric fruits, blackberry, Andean blueberry and goldenberry, were harvested at the final stage of edible maturity: stage 6 for blackberry [42] and Andean blueberry [32] and stage 5 for goldenberry. MI reached 9.64, very close to the value MI of 10.45 reported by Fischer et al. (2011) [35].

Changes during the postharvest period can modify firmness and color of fruits [13]. As can be seen in Table 2, naranjilla and tamarillo continued their ripening during storage and transportation, something related to their high variability observed in firmness of 36.52% and 29.69% respectively. Blackberry and araza suffered a firmness loss due to mechanical damages during transport. This data is not shown in the text. High water content and soft flesh make araza very susceptible to damage. Moreover, its exceptionally high respiration rate can change dramatically its color and firmness in a period as short as 72 h after being harvested [43]. In the same way, goldenberry presented a high variability in its firmness—14.72% at maturity—that could be attributed to being harvested with different MIs. This did not happen in the case of Andean blueberry.

### 3.2. Internal Color

**Figure 1:**
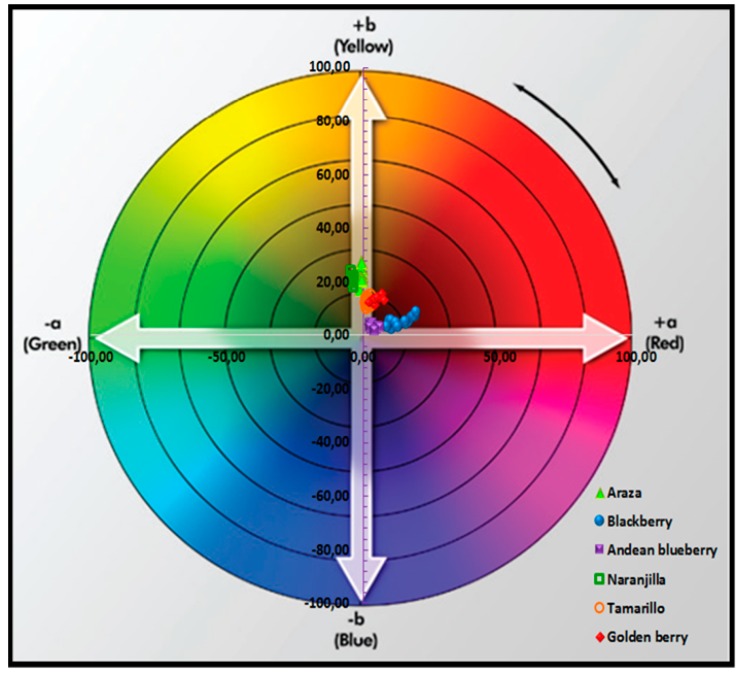
Chromatic representation of the CIE L*a*b* color coordinates of the tropical and Andean fruits.

The relationship between color and ripeness is due to the pigment accumulation and variation of the sugar and organic acid in fruits [44]. In Table 3 and Figure 1, it can be observed the internal color of the fruits was represented in the CIE L*a*b* color space. Araza and naranjilla pulp presented a clear trend towards green and yellow colors with an intermediate luminosity L* 49.63 and 40.10, respectively. Tamarillo and goldenberry presented a more yellow-reddish color and more differences in luminosity L*, 51.71 and 35.70, respectively. Blackberry and Andean blueberry presented the lowest values for luminosity L* 10.68 and 20.80, respectively, according to Hue et al., 2014 [17].

The rupture of the chlorophyll and accumulation of carotenoids is a phenomenon that occurs in araza, naranjilla, tamarillo and goldenberry. This phenomenon determines its color at ripeness, and turns the color of these fruits from green to yellow. β-carotene is the center of this photosynthetic reaction, its concentration in fruits being related to variety, harvest time and other factors like soil, climate, cultural practices, etc. [13]. In the case of blackberry and Andean blueberry, the color was dominated by red and blue, due to the characteristic accumulation of anthocyanins during ripening of these fruits [45].

All fruits evaluated presented polyphenols in their composition, Andean blueberry and blackberry being the fruits with the highest concentration of this antioxidant.

### 3.3. Development of the Mathematical Prediction Models

Correlation matrices were constructed using color coordinates L*, a* and b* as independent variables and TAC, TPC and TCC as dependent variables. Anthocyanin and polyphenol content models were developed for blackberry and Andean blueberry, whereas carotenoid and polyphenol content models were developed for araza, naranjilla, tamarillo and goldenberry.

Mathematical equations were evaluated considering two aspects, significance of the coefficients of the independent variables of the model and the global determination coefficient of the model. The mathematical equations were evaluated considering two aspects: significance of the coefficients of the independent variables of the model and the global determination coefficient of the model. The whole set of coordinates was used in order to elaborate the mathematical prediction models for color, as there were no significant differences for the partial coefficients of each coordinate L*, b*, and b* in terms of anthocyanin, carotenoid and total polyphenol contents.

The first modelling approach with the whole data resulted in very low determination coefficients for the TAC and TCC of all fruits. Based on the analysis of residuals between the theoretical and experimental data, the results obtained were consistent series of data falling inside a confidence interval of two standard deviations from the mean.

The selected data was submitted to a new multivariate analysis. The adjusted prediction equations for each fruit (Table 4; Table 5) were determined. The TAC prediction models had determination coefficients R^2^ of 0.84 and 0.98, whereas the TPC presented R^2^ between 0.81 and 0.89.

From the equations in Table 4, a prediction of bioactive compounds for each fruit was calculated in terms of TAC, TCC, and TPC. This result was compared to the experimental and bibliographic data in Table 5. The developed method of analysis was validated using the Horwitz variation coefficient (CV Horwitz). This parameter relates the concentration of analyte with the coefficient of variation of the experimental data. CV Horwitz was proposed as a reference value to evaluate the inter-laboratory tests performance. It has been accepted by the EU, IUPAC and CODEX to validate analytical methods. The value of CV Horwitz should be ≤16.0% [46].

### 3.4. Total Anthocyanin Content (TAC)

Anthocyanins were reported in mg/100 g accepting an error of 5–8%. As can be seen in Table 5, blackberry presented a TAC of 1416.68 ± 158.71 mg/100 g, DW, very close to the 637–3000 mg/100 g interval reported for different blackberry cultivars and harvesting conditions. The prediction model for blackberry showed a value of 1413 mg/100 g very close to the experimental data and with a coefficient of variation estimated (CV_E_) of 11.20.

Andean blueberry showed higher values of TAC of 2682.30 ± 602.92 mg/100 g close to data reported by Vasco et al. (2009) [47] around 3832.95 mg/100 g DW. The prediction model showed a good approximation to experimental data, offering a value of 2761.24 mg/100 g. In this case, the coefficient of variation predicted (CV_P_) of 2.66 was lower to the experimental error of CV_E_ of 5.74.

Blackberry and Andean blueberry showed red and violet dominance, correlated significantly with TAC, obtaining R^2^ values of 0.82 y 0.81, respectively.

Anthocyanins are part of a group of bioactive compounds present in the pulp of Andean blueberry and blackberry, and responsible for their characteristic red-blue color. In the case of araza, naranjilla, tamarillo and goldenberry, the presence of these chemical compounds was not identified, or their concentrations were under the detection limit of the spectrophotometric method employed. However, significant amounts of carotenoids were found in these fruits with characteristic yellow-orange color, as shown in Section 3.6.

### 3.5. Total Polyphenol Content

Polyphenols were reported in mg/100 g accepting an error of 5–8%. Polyphenols showed a significant correlation with the color of the six fruits studied, and especially those with red and violet colors, obtaining coefficients of 0.88 for araza, 0.81 for blackberry, 0.82 for mortiño, 0.84 for naranjilla, 0.75 for tamarillo and 0.88 in goldenberry.

As can be seen in Table 6, Andean blueberry showed the highest TPC, 7254.62 ± 1209.17 mg/100 g; CV_E_ 10.86%. These results are in accordance with the ones reported by several authors working with Ecuadorian Andean blueberry of 8104.52–9799.02 mg/100 g [48]. The prediction models showed a value of 7315.73 mg/100 g with a CV_P_ of 2.22%.

Blackberry showed the second highest TPC of 6352.28 ± 633.61 mg/100 g; CV_E_ 4.47% falling into the upper part of the interval 2340.24−6300 mg/100 g reported by several authors [49]. The prediction model showed a theoretical value for this fruit of 5995.62 mg/100 g, with a CV_P_ of 3.38% (Table 5).

Araza also showed a high TPC of 3507.79 ± 1430.36 mg/100 g with a CV_E_ 4.65% in accordance to the results reported by Laverde-Acurio, (2010) [50] for clone 003 (2477.72 mg/100 g). In this case, the predicted value for TPC (3256.33 mg/100 g) resulted in a CV_P_ of 0.98% (Table 5).

Tamarillo from ecotype Orange Giant showed a TPC of 1062.77 ± 57.87 mg/100 g; with a CV_E_ 10.26%. Torres (2006) [51] showed a value for this fruit of 654.20 mg/100 g DW. The predicted value from equations obtained resulted in 1055.45 ± 14.80 mg/100 g with a CV_P_ 0.72% (Table 5).

Naranjilla cv. INIAP quitoense 2009 showed a TPC of 897.58 ± 227.77 mg/100 g and a CV_E_ of 6.32%, above the 510.72–699.79 mg/100 g interval reported by several authors for several agroclimatic conditions [51]. The predicted value for TPC in naranjilla was 730.84 mg/100 g, with a CV_P_ of 2.02% (Table 5).

Experimental TPC obtained for goldenberry was 259.93 ± 42.74 mg/100 g with a CV_E_ 11.61%, whereas the predicted TPC resulted in 233.68 mg/100 g, with a CVP of 3.24%. Both results are lower than bibliographic data reported by Cerón et al. (2010) in goldenberry grown in Colombia [52].

Experimental errors for Andean blueberry of 10.86%, tamarillo with 10.26% and goldenberry with 11.61% were slightly higher than the upper limit imposed by Horwitz criterium for this range of concentrations, 5–8%.

### 3.6. Total Carotenoid Content

Carotenoid concentration was measured in µg/g, accepting an error of <16%. Table 5 shows the TCC results: tamarillo showed a dry basis TCC of 123.18 ± 16.61 µg/g with a CV_E_ 4.65%, the highest concentration of TCC among all fruit studied and very similar to data reported by Mertz et al. (2009) [45] for the same cultivar and origin of 117.37 µg/g. The prediction model showed a TCC of 133.67 µg/g with a CV_P_ of 0.98%. Both CV were well above the Horwitz limit for this range of concentrations around 16%.

Goldenberry showed a TCC of 65.21 ± 8.31µg/g with a CV_E_ 10.84% very close to data reported by Ramadan, (2011) who obtained a TCC of 85.38 µg/g on a dry basis for the ecotype Golden Kenyan. The prediction value for this fruit of 64.93 µg/g showed an estimated error of 6.49% also lower than the Horwitz limit [30].

In araza, an experimental TCC of 62.85 ± 3.36 µg/g with a CV_E_ 1.92% and a predicted value of 61.96 µg/g with a CV_P_ 1.09% were found. These results are close to 55.32 µg/g, DW, in accordance with the ones reported by Laverde-Acurio (2010), in clone 003 [50].

Finally, naranjilla (var. INIAP quitoense 2009) showed the lowest concentration of experimental TCC, 57.93 ± 4.28 µg/g with a value of CV_E_ 6.05% and a predicted a value of 58.42 µg/g, with a value of CV_P_ 4.50%, relatively close to data reported by Acosta et al. (2009) [34] in naranjilla from Costa Rica of 76.59 µg/g, DW.

The results obtained for araza, naranjilla, tamarillo and goldenberry confirmed that TCC found in these fruits are strongly associated with color parameters L*, a* and b* and thus being responsible for the yellow-orange color of the pulp of these fruits, obtaining coefficients R^2^ ranged from 0.85 to 0.98 for the latter fruits. Also, it is worth noting that all experimental and predicted values for TCC were within the threshold reported by Horwitz for the concentration range of µg/g with a value of CV Horwitz 16%, as commented before.

**Table 4:** Mathematical prediction models for total anthocyanin content (TAC), total carotenoid content (TCC) and total polyphenol content (TPC) of six tropical and Andean fruits from Ecuador.

Fruits	*TAC* = *b*_0_ + *b*_1_(L*) + *b*_2_(a*) + *b*_3_(b*)					*TCC* = *b*_0_ + *b*_1_(L*) + *b*_2_(a*) + *b*_3_(b*)					*TPC* = *b*_0_ + *b*_1_(L*) + *b*_2_(a*) + *b*_3_(b*)				
	b_0_	b_1_	b_2_	b_3_	R^2^	b_0_	b_1_	b_2_	b_3_	R^2^	b_0_	b_1_	b_2_	b_3_	R^2^
Blackberry	1644.47	−13.53	−12.56	18.42	0.82	N. D	N. D	N. D	N. D	N. D	8375.00	−41.15	−132.30	18.79	0.82
Andean blueberry	−3206.38	233.19	96.97	264.9	0.81	N. D	N. D	N. D	N. D	N. D	7095.72	33.44	143.50	−308.63	0.82
Naranjilla	N, D	N. D	N. D	N. D	N. D	129.24	−1.04	1.47	−0.98	0.84	1151.18	−15.69	−74.94	−5.58	0.84
Tamarillo	N. D	N. D	N. D	N. D	N. D	79.29	−1.12	3.93	3.35	0.98	1247.55	−5.53	53.00	−11.51	0.75
Goldenberry	N. D	N. D	N. D	N. D	N. D	−24.31	2.19	2.41	−0.23	0.85	−142.76	9.85	10.09	−1.91	0.88
Araza	N. D	N. D	N. D	N. D	N. D	83.52	−0.84	−5.88	0.7	0.93	−4974.64	381.95	932.80	−450.89	0.88

TAC: total anthocyanin content; TCC: total carotenoid content; TPC: total polyphenol content and N.D: non- detectable.

**Table 5:** Experimental and predicted values for total anthocyanin content (TAC), total carotenoid content (TCC) and total phenolic content (TPC) of six tropical and Andean fruits from Ecuador and a comparison with data reported in literature.

Fruit	Antioxidant Compounds														
	* (TAC)					* (TCC)					* (TPC)				
	(mg cyanidin 3-glucoside chloride/100 g)					(µg β-carotene/g)					(mg GAE/100 g)				
	Experimental		Predicted		Bibliographic	Experimental		Predicted		Bibliographic	Experimental		Predicted		Bibliographic
	Mean	CV_E_	Mean	CV_P_		Mean	CV_E_	Mean	CV_P_		Mean	CV_E_	Mean	CV_P_	
Blackberry	1416.69	11.20	1413.00	0.85	637–3000 [27,46]	N. D					6352.28	4.77	5995.62	3.38	2340.24−6300 [27,46,53]
Andean blueberry	2682.30	2.66	2761.24	5.74	3832.95 [48]	N. D					7254.62	10.86	7315.73	2.22	8104.52−9799.02 [48,54]
Naranjilla	N. D					57.93	6.05	58.42	2.36	76.59 [55]	897.58	6.32	912.50	2.02	510.72−699.79 [49,50,53]
Tamarillo	N. D					123.18	4.65	133.67	1.95	117.37 [46]	1062.77	10.26	1055.45	0.72	654,20 [52]
Goldenberry	N. D					65.21	10.84	64.93	3.10	85.30 [30]	259.93	11.61	233.68	3.24	215.60 [34]
Araza	N. D					62.85	1.92	61.96	1.09	55.32 [51]	3507.79	13.97	3256.33	6.94	[51]

* Results are reported as dry basis ± standard deviation (*n =* 45) for each fruit. N.D: non-detectable; GAE: gallic acid equivalents; CV_E_: coefficient of variation estimated; CV_p_: coefficient of variation predicted.

### 3.7. Antioxidant Activity Using ABTS and DPPH Methods

The extracts obtained from araza ((*Eugenia stipitata* McVaugh), tamarillo (*Solanum betaceum* Cav.) and naranjilla (*Solanum quitoense* Lam.) were used to evaluate their antioxidant activity using the ABTS and DPPH methods. Araza fruit presented a higher value of ABTS and DPPH with 758.22 µmoL TE/g sample and 392.10 µmoL TE/g, sample respectively. Tamarillo presented an ABTS value of 161.04 µmoL TE/g sample and 47.82. µmoL TE/g sample (Table 6). The value of ABTS and DPPH between fruits presented statistical differences at *p* < 0.05. Naranjilla fruit presented a low value of antioxidant activity with both methods. Espin et al. (2016) described the antioxidant activity of four varieties of tamarilllo fruits (*S. betaceum* Cav.) using the ABTS, FRAP and ORAC methods. ABTS analysis presented a value between 22 and 89 µmoL TE/g of the sample, lower than the one reported here [54]. It can be explained by the fact that the fruits used in our study were obtained from different cultivars and locations in Ecuador. Araza fruit has a high antioxidant activity with both methods and can be related to its high TPC content of 3507.79 mg GAE/100 g DW because it does not register TAC and its content of TCC was low; i.e., the experimental value of 62.81 µg β-carotene/g and 61.96 µg β-carotene/g predicted by the mathematical model.

**Table 6:** Antioxidant activity of araza, tamarillo and naranjilla fruits using the ABTS and DPPH methods.

Fruit	Antioxidant Activity	
	(µmoL TE/g Sample)	
	ABTS Assay	DPPH Assay
Araza	758.22 ± 5.01 ^a^	392.10 ± 9.67 ^a^
Tamarillo	161.04 ± 8.48 ^b^	47.82 ± 2.94 ^b^
Naranjilla	76.40 ± 1.33^c^	21.26 ± 1.35 ^c^

The ABTS and DPPH results were presented as the means ± standard deviation. Values in the same column followed by different letters are statistical meaningful at *p* < 0.05 using the ANOVA-one-way analysis followed by the Tukey’s Test.

Foods are complex matrices that have many biomolecules and secondary metabolites that may or may not be reactive to the available antioxidant methods. It must be borne in mind that the content of these bioactive compounds in fruits can be modified by the environmental conditions of the crop [56,57]. Therefore, it is important to know the nature of the sample and try to separate the components as much as possible before choosing the method. It would also be appropriate to measure with different methods to look for differences [58,59]. The different bioactive compounds can be separated and identified with different analytical techniques. For example, antioxidant and phenolic compounds can be separated and identified using high performance liquid chromatography (HPLC) coupled with a post column derivatization (PCD). The analyses allow to relate changes in the chemistry of the state of the food directly with the presence or absence of the detected compounds. Another form of analysis of antioxidant and/or polyphenols is to fractionate the sample by semi-preparative HPLC and subsequently test the antioxidant activity of the fractions collected [60]. However, the HPLC-PCD technique is a faster technique and may be more effective for the detection and identification of these bio compounds [61]. Nonetheless, the mathematical model presented in this study for the first time reports that the TAC, TCC and TPC predictions in six fruits of Ecuador, serving as a first screening method. It would be necessary to validate the mathematical model in other fruits to see its effectiveness in order to determine the TAC, TCC, and TPC in other fruits with different qualitative and quantitative phenolic/carotenoid composition. This method can be used as a first screening to determine the TAC, TCC and TPC content but cannot replace an HPLC analysis of the individual components.

## 4. Conclusions

The mathematical prediction models developed allowed the determination of the TAC, TCC and TPC in araza, Andean blueberry, goldenberry, naranjilla, tamarillo and blackberry in a fast, precise and non-destructive way. These models allow reducing the cost of analysis compared to traditional chemical methods, avoiding the use of solvents and other materials for sample preparation. These satisfactory mathematical models resulted in a prediction of the TAC content of blackberry and Andean blueberry. The TCC was successfully predicted for araza, naranjilla, tamarillo and goldenberry, whereas the TPC was correctly predicted for araza, blackberry, Andean blueberry, tamarillo and goldenberry. The models showed a good statistical calibration as a result of their high determination coefficients R^2^, ranging between 0.75 and 0.98. There is a high correlation between experimental and predicted data from the mathematical models. The prediction errors obtained ranged between 0.72% and 6.94% and were kept within the limits according to the Horwitz criterium CV of 5–16%. This method can be used as a routine system susceptible of being evaluated using laboratory systematic methods.

## Figures and Tables

**Table 1:** Multivariate components matrix.

Biocompounds Content	Luminosity	Coordinate Red/Green	Coordinate Yellow/Blue
	(L*)	(a*)	(b*)
Y_1_	X_11_	X_12_	X_13_
Y_2_	X_21_	X_22_	X_23_
Y_n_	X_n1_	X_n2_	X_n3_
Mean	X_1_	X_2_	X_3_

**Table 2:** Physical and chemical parameters related to the maturity stage of the fruits.

Sample	Titrable	Total Soluble Solids *	Maturity	Firmness *
	Acidity *	(°Brix)	Index *	(N)
	(%)			
Araza	2.40 ± 0.02	3.83 ± 1.19	1.60 ± 0.45	14.42 ± 5.00
Blackberry	2.81 ± 0.07	12.69 ± 0.43	4.51 ± 0.06	3.22 ± 0.49
Andean blueberry	0.96 ± 0.05	11.81 ± 0.26	12.39 ± 0.72	0.69 ± 0.01
Naranjilla	2.58 ± 0.15	9.55 ± 0.43	3.72 ± 0.32	48.05 ± 17.55
Tamarillo	2.09 ± 0.05	12.43 ± 0.94	5.94 ± 0.37	55.80 ± 16.57
Goldenberry	1.42 ± 0.02	13.73 ± 1.50	9.64 ± 0.62	2.65 ± 0.39

* Fresh basis.

**Table 3:** CIE L*a*b* color coordinates in several tropical and Andean fruits.

Fruit	Color Coordinates							
	Lightness		Red-Green		Blue-Yellow		Chroma	Hue
	L*	CV	a*	CV	b*	CV	C*	°H
		(%)		(%)		(%)		
Araza	49.63 ± 2.94	5.91	−0.89 ± 0.40	45.32	22.73 ± 2.84	12.48	92.31 ± 1.20	22.75 ± 2.83
Blackberry	10.68 ± 2.23	20.88	13.80 ± 3.90	28.26	4.95 ± 1.70	34.34	19.86 ± 4.94	14.71 ± 27.85
Andean blueberry	20.80 ± 1.50	7.21	3.52 ± 1.10	31.25	3.16 ± 0.95	30.06	42.28 ± 13.38	4.86 ± 0.87
Naranjilla	40.10 ± 1.92	4.79	−4.25 ± 0.60	14.03	22.04 ± 2.62	11.88	100.93 ± 1.05	22.45 ± 2.65
Tamarillo	51.75 ± 2.93	5.67	9.06 ± 0.71	7.84	32.68 ± 2.83	8.65	74.45 ± 1.41	33.92 ± 2.80
Goldenberry	35.70 ± 2.46	6.88	7.10 ± 0.51	7.25	25.39 ± 3.55	13.99	74.11 ± 2.48	26.39 ± 3.41
